# Novel Technique for the Surgical Management of Pleomorphic Adenoma of the Upper Lip

**DOI:** 10.7759/cureus.27214

**Published:** 2022-07-24

**Authors:** Dyna Albert, Senthil Murugan Pandurangan, Purva Kulkarni, Santhosh P Kumar, Murugesan Krishnan, Twinkle Francis

**Affiliations:** 1 Oral and Maxillofacial Surgery, Saveetha Dental college and Hospital, Chennai, IND; 2 Oral and Maxillofacial Surgery, Saveetha Dental College and Hospital, Chennai, IND

**Keywords:** mixed tumor, benign tumor, surgical management, innovative technique, novel technique, upper lip reconstruction, minor salivary gland tumor, upper lip, benign pleomorphic adenoma

## Abstract

Pleomorphic adenoma (PA) is one of the most common benign lesions of the salivary glands, with a majority of them occurring in the parotid gland. PA has origin from the epithelial and mesenchymal elements and can arise from both major and minor salivary glands. Among minor salivary glands, the palate is the most commonly affected site, followed by lips, cheeks, gingiva, the floor of the mouth, and tongue. PA of the upper lip without intraoral mucosal involvement is a rare entity. In this article, we report a case of PA of the upper lip in a middle-aged female patient and its surgical management with a novel technique. During six months’ postoperative review, the patient showed excellent wound healing with very minimal scar formation.

## Introduction

Pleomorphic adenoma (PA) is a benign mixed tumor of the salivary gland that has origin from both the epithelial and mesenchymal elements. PA constitutes 45-75% of all salivary gland tumors and 70-80% of benign salivary gland tumors [1]. It most commonly occurs in the parotid gland (84%) followed by the submandibular gland (8%) and minor salivary glands (6.5%). Intra-orally, it can occur in the palate, buccal mucosa, retromolar area, upper lip, and floor of the mouth, with the palate (both the hard and soft palate) being the most common site of occurrence [2]. Although it can affect both genders and occur in any age group, it is more common in females than in males (2:1) and occurs more frequently in the third to sixth decades of life [3]. Clinically, it manifests as a solitary, mobile, painless, slow-growing tumor of longer duration. The term “pleomorphic” means “variable in appearance” and depicts the histological pleomorphism of the tumor [4]. Microscopically, they show mixed proliferation, which is characterized by spindle-shaped myoepithelial and polygonal epithelial cells in a variable stroma that is of hyaline, mucoid, myxoid, or cartilaginous origin [5].

## Case presentation

**Figure 1:**
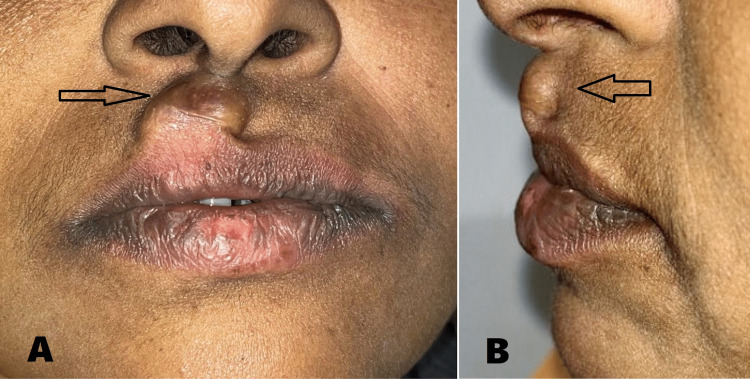
Extraoral view showing lesion (arrow) on the right side of the upper lip. (A) Frontal view. (B) Profile view.

**Figure 2:**
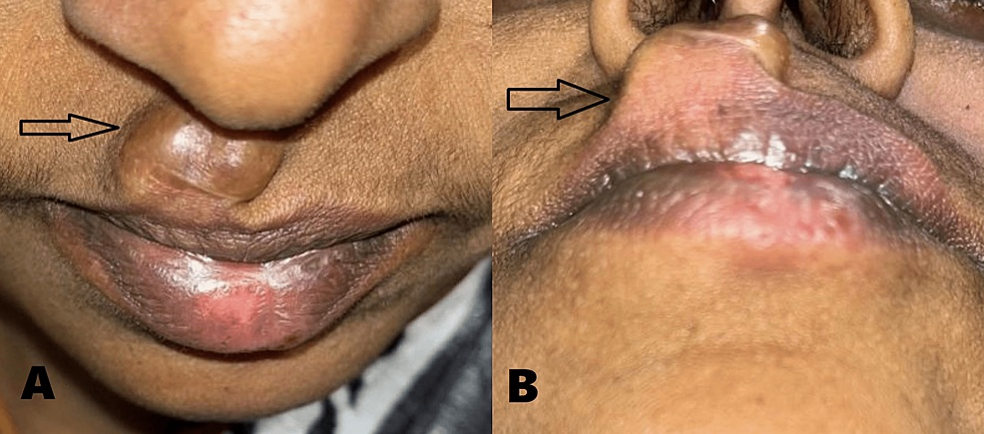
Extraoral view showing lesion (arrow) on the right side of the upper lip. (A) Bird’s-eye view. (B) Worm’s-eye view.

A 46-year-old female presented to the Department of Oral and Maxillofacial Surgery with a chief complaint of painless swelling in the upper lip for the past five years. History revealed that the growth was initially small in size and gradually increased to attain the present size. The patient gave no relevant past medical or surgical history. On examination, the lesion was located in the extra-oral aspect of the upper lip on the right side, and there was no intraoral swelling (Figure 1). It was well-circumscribed, lobulated into two discrete swellings, measuring 10 x 8 mm in dimension and extending superoinferiorly from sub-nasale to vermillion of upper lip including the peak of cupids bow on the right side. Medially, the swelling involved the right philtral ridge and did not cross the left philtral ridge. Laterally, it did not extend beyond the width of the right-side nasal sill, and there was an evident vermillion mismatch due to the underlying lesion (Figure 2). It was soft to firm in consistency, non-tender, yielded to pressure, and elicited a positive slip sign. The skin over the swelling was shiny and relatively darker than the surrounding tissues.

Differential diagnoses of the lesion included PA, epidermoid cyst, sebaceous cyst, and lipoma. The patient was advised ultrasonography (USG), which was suggestive of a sebaceous cyst measuring 1.4 x 0.7 cm on the right upper lip. Surgical excision of the lesion and reconstruction of the upper lip based on the principles of modified Millard’s rotation advancement flap was planned under general anesthesia.

**Figure 3:**
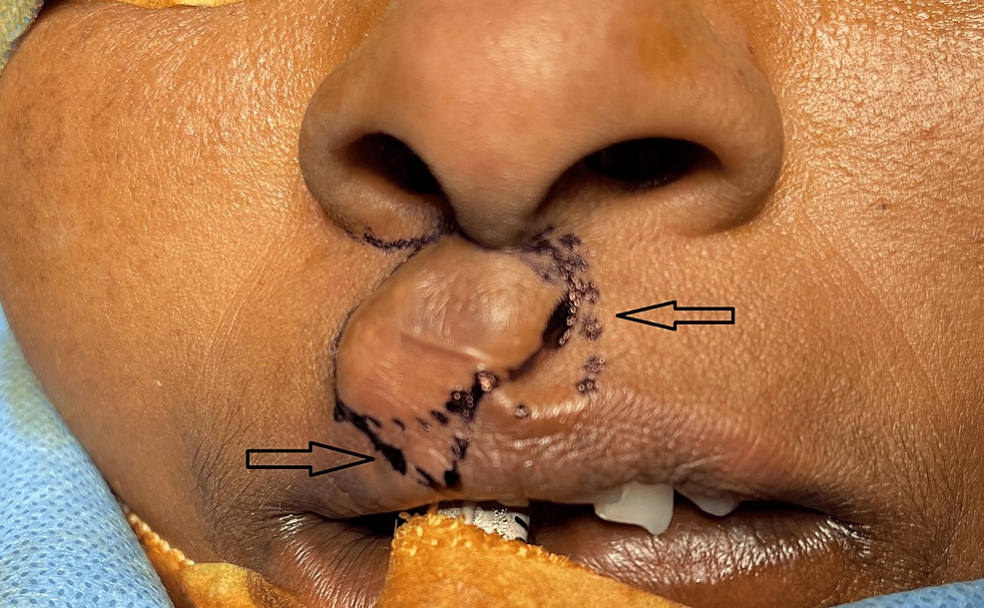
Markings (arrows) for surgical excision and closure using Millard’s principle of rotation advancement flap.

**Figure 4:**
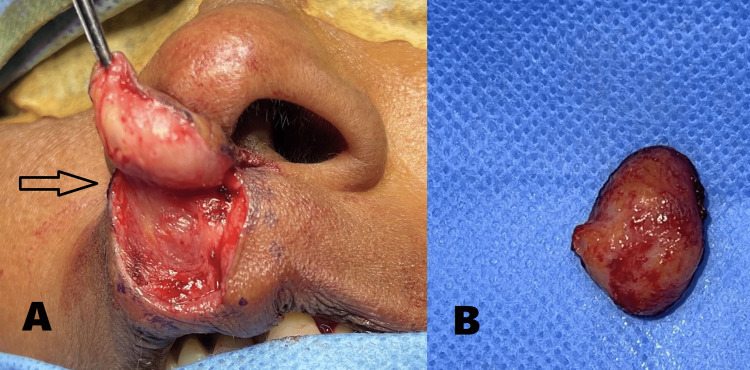
(A) Lesion dissected (arrow) and freed from the surrounding tissues. (B) Excised specimen In toto with the overlying skin.

**Figure 5:**
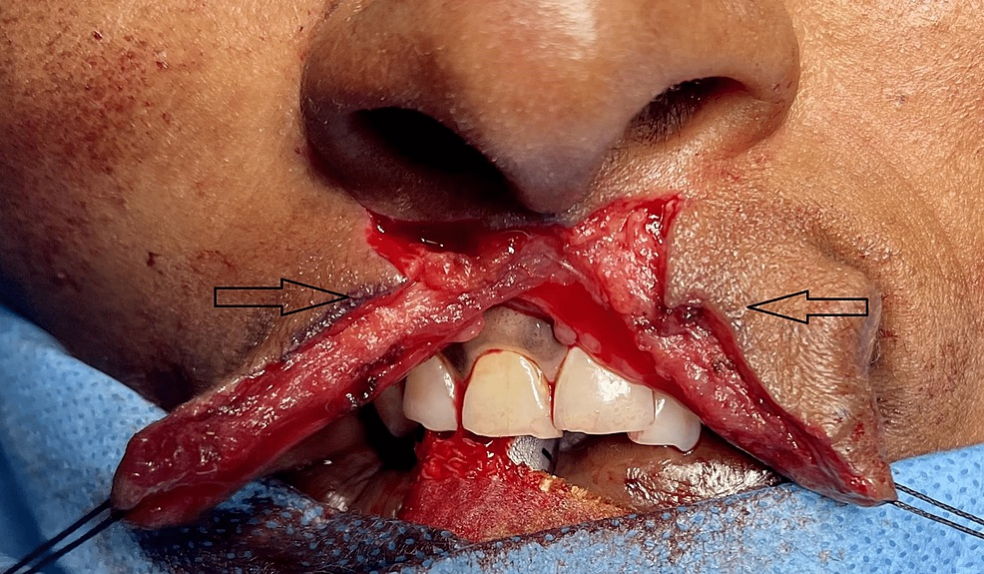
Incision and dissection performed (arrows) based on Millard’s rotation advancement flap technique for reconstruction of the upper lip defect.

Local anesthesia was infiltrated at surgical sites on the upper lip. Markings were made around the lesion and involved the philtral column on the non-lesion side also (Figure 3). The lesion was dissected and freed from the surrounding tissue,s and the specimen was excised in toto from its base with the overlying skin (Figure 4). Markings, incision, and dissection were performed based on the principles of modified Millard’s rotation advancement flap technique for surgery of cleft lip. Back cuts were given on both sides at the base of the columella and were followed by through and through dissection involving the vermillion border (Figure 5).

**Figure 6:**
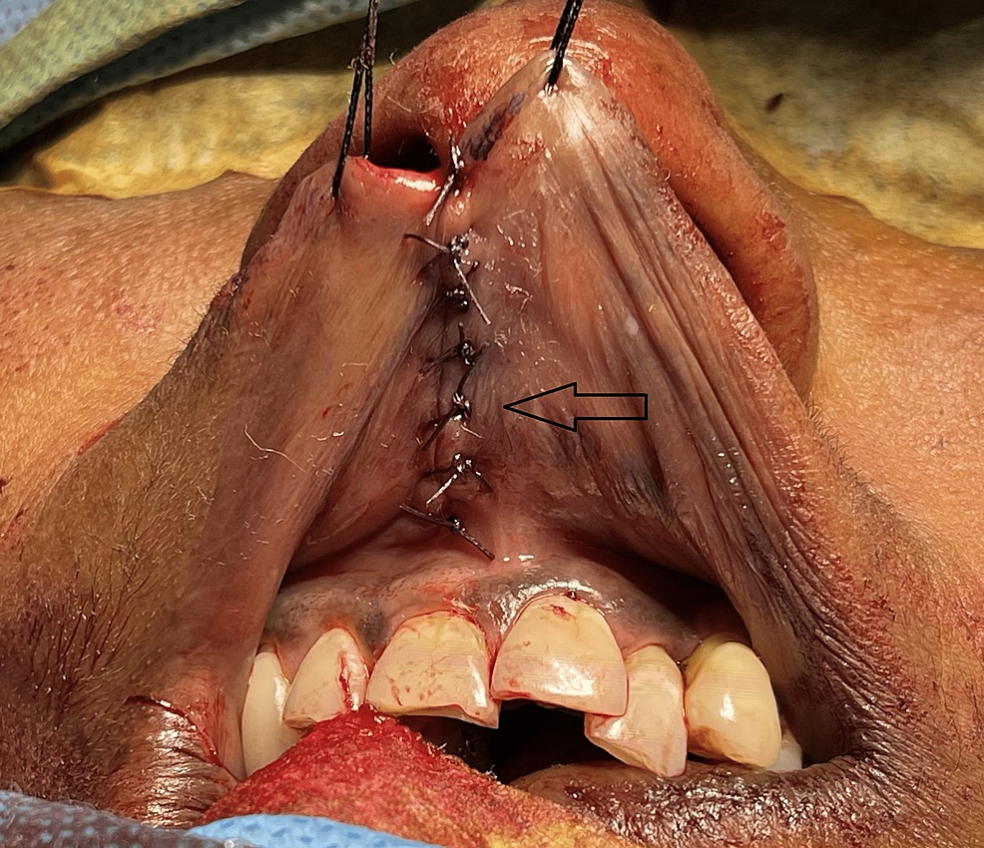
Closure of the oral mucosal layer (arrow).

**Figure 7:**
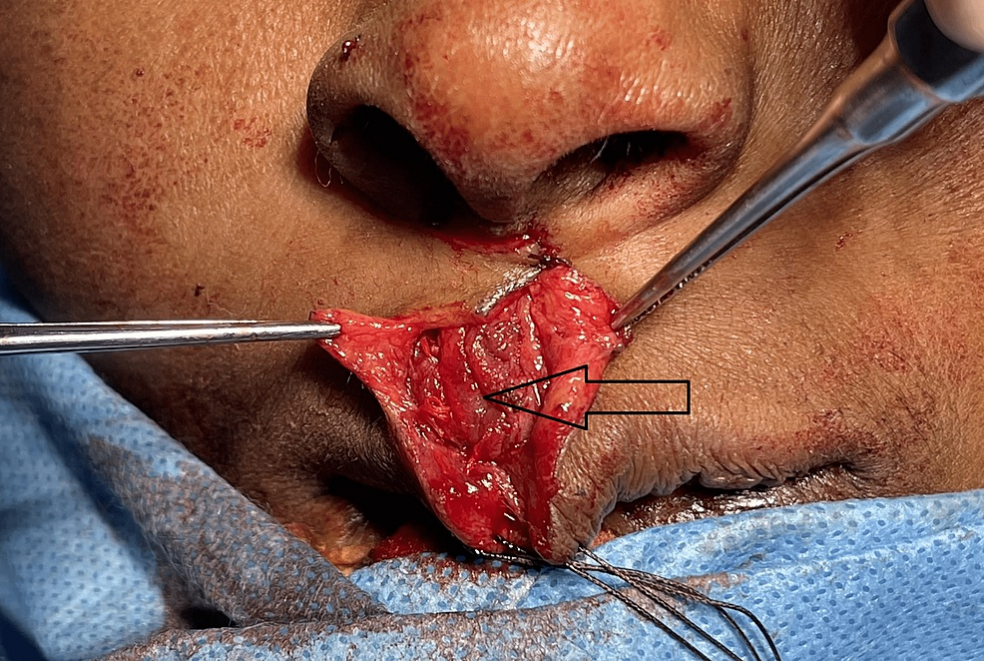
Closure of the muscle layer (arrow).

**Figure 8:**
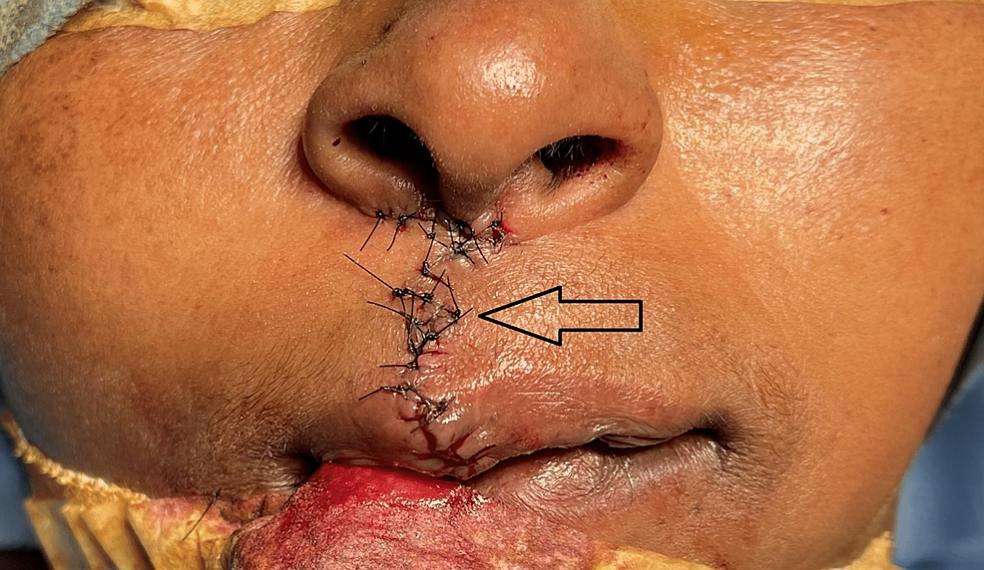
Closure of the skin layer (arrow).

The underneath muscle, orbicularis oris, was resected. Closure of the oral mucosal layer was performed with resorbable sutures (Figure 6). Re-approximation and closure of the muscle layer in three planes were performed with resorbable sutures (Figure 7). Vermillion mismatch was corrected, and the final linear closure of the skin was performed using non-resorbable sutures (Figure 8).

**Figure 9:**
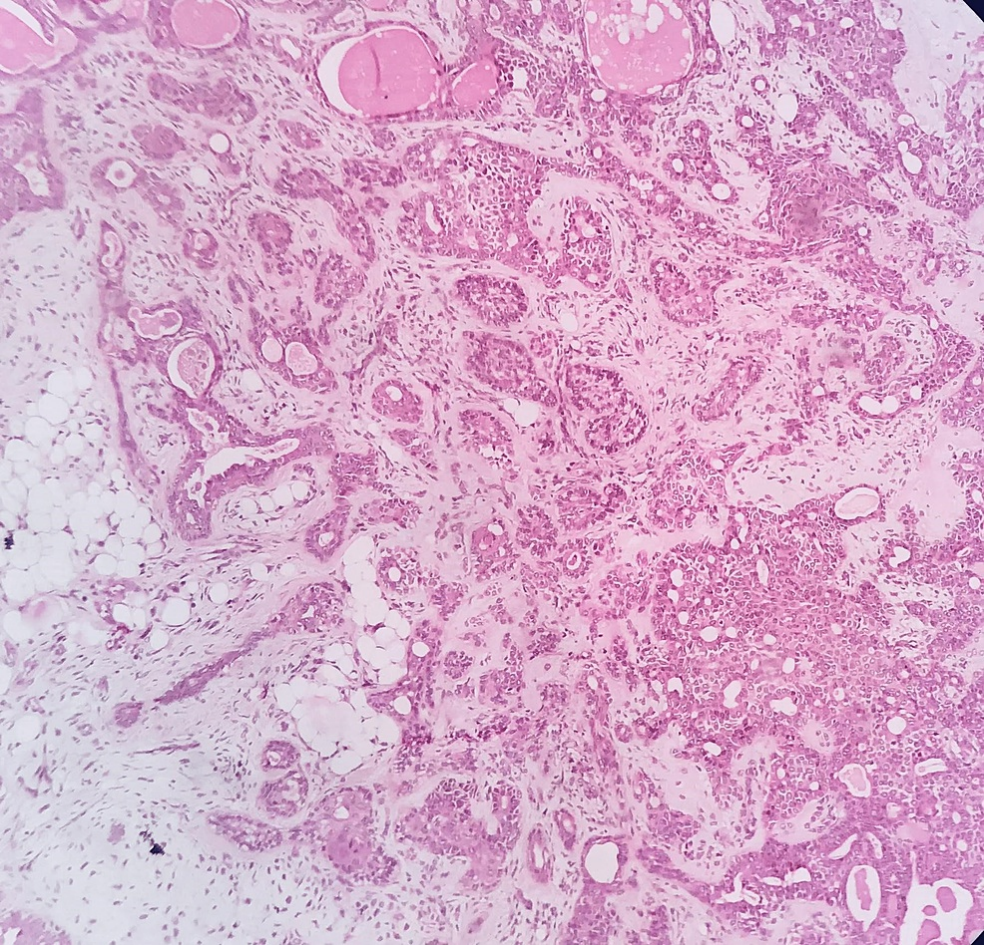
Histological examination shows features typical of pleomorphic adenoma (hematoxylin and eosin stain, 100× magnification).

**Figure 10:**
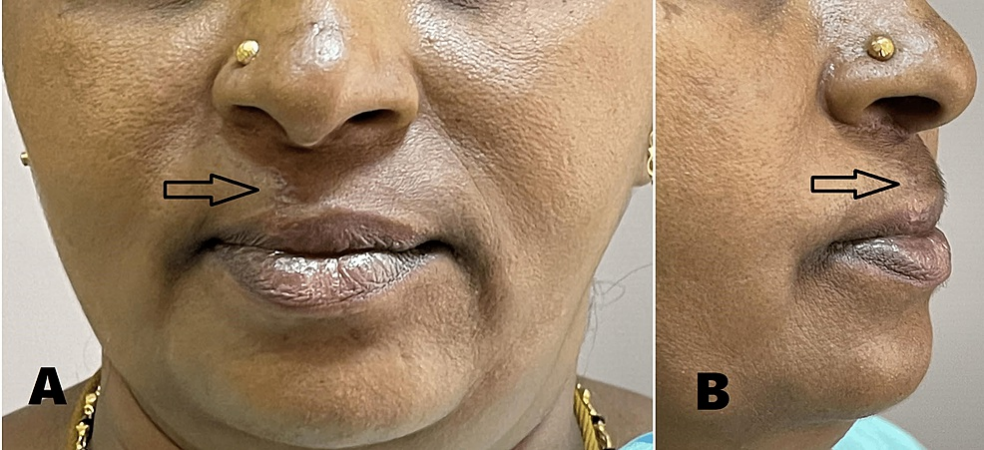
Six months’ postoperative clinical picture showing excellent wound healing (arrows). (A) Frontal view. (B) Profile view.

Histopathological examination of the specimen showed a non-encapsulated neoplasm of glandular epithelium admixed with myxoid connective tissue stroma. The epithelial component was composed of tumor cells arranged in the form of duct areas of varying sizes and shapes, with a central lumen containing an eosinophilic coagulum lined by cuboidal cells. These duct-like areas were surrounded by angular, spindle cells merging into the myxoid connective tissue stroma. There were sheets of numerous rounded to oval cells with an eccentrically placed nucleus and eosinophilic cytoplasm suggestive of plasmacytoid cells. Several areas showed hyalinization with squamous and adipocytic metaplasia. The connective tissue stroma showed myxoid-like areas along with moderate vascularity and areas of hemorrhage. The overlying epithelium showed ortho-keratinized stratified squamous epithelium of variable thickness suggestive of dermal epithelium with dermal appendages. Thus, the findings confirmed the diagnosis of PA of the salivary gland (Figure 9). During six months’ postoperative review, the patient showed excellent wound healing with very minimal scar formation (Figure 10).

## Discussion

PA is one of the most common benign lesions of the salivary glands, with a majority of them occurring in the parotid gland. PA also arises from the intraoral minor salivary glands mostly involving the hard palate and soft palate. PA of the lip is common compared to other regions such as the cheek, floor of the mouth, retromolar trigone, and the gingiva [6]. In our case, PA of the minor salivary gland occurred around external cutaneous skin without intraoral mucosal involvement in the upper lip region and is a rare entity. The lesion was circumscribed, and a definite vermillion mismatch was seen with color discoloration over the skin involving the lesion. Clinical features were suggestive of a benign pathology, and the lesion resembled a sebaceous cyst. Differentials diagnosis of the lesion included PA, lipoma, subdermoid cyst, sebaceous cyst, irritation fibroma, salivary gland duct cyst, and tumor.

The upper lip is the second most common site for the occurrence of PA in the intraoral minor salivary glands [7-10]. PA is more common in the upper lip than in the lower lip because of the complex embryological development of the upper lip compared to the lower lip [11]. The upper lip is usually associated with benign lesions, and malignant lesions are more frequently seen in the lower lip [12]. In our case, the patient had no history of previous salivary gland pathology. The age and gender of the patient and clinical features were indicative of benign pathology of the minor salivary gland. Mild discoloration on the overlying skin of the lesion and its erosive nature leading to a mismatch of the vermillion border ruled out the lesion being an irrational fibroma or lipoma. Though USG suggested the lesion to be a sebaceous cyst, a benign pathology of the minor salivary gland was suspected due to the mixed clinical presentation.

The treatment plan was in toto surgical excision of the lesion followed by histopathological examination. The lesion was excised along with the overlying skin, providing a clearance of 5 mm and maintaining the integrity of the capsule. Surgical excision is the primary treatment for salivary gland neoplasms. For malignant lesions, a margin of 1-2 cm should be excised along with the lesion [13]. Minor salivary gland tumors have a recurrence rate of 5-30% if surgical removal is inadequate, and malignant neoplasms have a higher recurrence rate of up to 65%. The tendency for recurrence of the tumor is mainly based on the histopathological characteristics of the tumor; thus, a clearance of 3-5 mm during surgical excision of benign pathologies is recommended [14,15].

In our case, the residual defect after excision was large, and primary closure would lead to gaping of the wound or result in an unsightly scar. To overcome this shortcoming and to achieve a tension-free closure, the rotation and advancement of Millard’s technique were adopted and modified to suit our case. Millard’s technique was followed and an intentional through and through incision of the upper lip was made, and back cuts were created to accommodate the C-flap [16]. This technique achieved tension-free closure and significantly reduced postoperative scar formation. The vermillion mismatch was also corrected and its integrity was re-established. This is a novel method of adopting Millard’s rotation advancement philosophy of cleft lip surgery in the surgical management of PA of the upper lip.

## Conclusions

PA arising from the minor salivary glands of the upper lip and not manifesting intraorally is a rare entity. Clinical diagnosis of salivary gland tumors related to oral mucosa is quite challenging. PA of the upper lip resembles several benign lesions, and final confirmation of the pathology is always based on the histopathological report. Proper surgical planning and execution are important in the management of PA in salivary glands, and long-term follow-up is recommended as they tend to recur. After surgical excision of the PA of the upper lip, Millard’s rotation advancement philosophy of cleft lip surgery can be utilized in the reconstruction of upper lip defects.
